# Development of a low-cost device for testing glove and condom leakage

**DOI:** 10.1016/j.amsu.2022.103791

**Published:** 2022-05-17

**Authors:** Mekides Abera Seyoum, Menen Teshome Belachew, Meron Berihun Dessale, Meti Wakjira Adugna, Tizita Yohannes Keto, Yezbalem Getnet Wassie, Habtamu Abafoge Dobamo, Melese Uma Getaneh, Hundessa Daba Nemomssa

**Affiliations:** a School of Biomedical Engineering, Jimma Institute of Technology, Jimma University, Jimma, Oromia, Ethiopia; b Gilando Biomedical Solution Plc, Addis Ababa, Ethiopia; c Jimma University Medical Center, Jimma University, Jimma, Oromia, Ethiopia

**Keywords:** Condom leakage, Glove leakage, Hospital acquired infection, Sexually transmitted diseases, Unwanted pregnancy, Low-cost tester, Resource constrained areas

## Abstract

**Background:**

Currently, hospital-acquired infections in healthcare workers and patients are a major concern. On the other hand, sexually transmitted infections and diseases, unwanted pregnancies, and unsafe abortions continue to be a public health concern, particularly in developing countries. Gloves are among the most commonly used personal protective equipment to safeguard healthcare workers' hands from contagious infections, and using a condom is strongly advised for people who have sexual relations with more than one partner. However, the quality of gloves and condoms in developing countries is a subject of concern. The usage of quality test instruments such as glove leakage test apparatus (GLTA), leakage testers by water level, the Ammonia leak testing method (ALTM), conductivity-based leakage testers, and water hang testers in developing countries is limited owing to cost, accessibility, and safety. The main purpose of this study was to develop and test a low-cost integrated device to test glove and condom leakage that is safe and easily accessible in resource poor settings.

**Method:**

In this study, an integrated glove and condom leakage testing device for detecting pin holes and leakages is proposed. The device automatically fills a randomly selected condom and glove with a predetermined volume of water based on International Organization for Standardization (ISO) criteria.

**Results:**

The prototype of the proposed device was successfully developed and tested. The accuracy of 98.66% for filling condom samples with 300 ml of water and 99.29% for filling glove samples with 1000 ml of water was achieved.

**Conclusion:**

The implementation of the developed prototype in resource poor settings to test gloves and condom leakage has the potential to improve the safety of healthcare workers, patients, and the general public.

## Highlights

• Hospital acquired infection to healthcare personnel and patients is a pressing issue.
• Sexually transmitted diseases and the associated problems continue to be a public health concern in developing countries.
• Gloves and condoms are among the most commonly used personal protective equipment.
• The quality of gloves and condoms in developing countries is a subject of concern to healthcare workers and general public.
• The automatic glove and condom leakage test device using water leakage test method for glove and condom test was proposed.

## 1 Introduction

With a number of contagious diseases surrounding the hospital setting, including the current COVID-19 pandemic, access to and usage of standard personal protective equipment (PPE) by healthcare workers (HCWs) is a critical issue today [[1], [2], [3], [4]]. Some of the PPEs used to protect and ensure the safety of HCWs from contagious diseases include aprons, long-sleeved gowns, gloves, respirators, and goggles [5,6]. Gloves, among other items, are the most commonly used PPE to protect HCWs' hands from contamination [7,8]. The effectiveness of gloves to prevent infections and contamination is dependent on their quality as well as the lack of leakage [9]. The integrity of a glove depends on several factors: material, brand, activity, and wearing duration [[10], [11], [12], [13]]. In the medical industry, glove leakage is more common on examination gloves than on surgical gloves [14]. A study showed that 5–10% of the hands of healthcare workers get contaminated after glove removal [15] which indicates that leakage in gloves could play a critical role in cross-transmission of pathogens [16,17]. According to the World Health Organization (WHO), 3 million percutaneous exposures occur annually among 35 million health care workers, with 90% of them occurring in poor resource settings [18].

On the other hand, condoms are used during intercourse to reduce unwanted pregnancy, sexually transmitted infections, and sexually transmitted diseases by creating a barrier between the sexual organs [19]. Low-quality condoms, which pose a great risk to end users, are reported in many countries as the main concern. The main issues are holes in condoms, tearing during use, and counterfeit branding. A study conducted in the Dominican Republic on ten condom brands indicates that five brands were found to have extensive quality problems, with 5.7%–17.5% of the condoms found to have holes [20].

Glove leakage test apparatus (GLTA) [21], leakage testers by water level [22], the Ammonia leak testing method (ALTM) [23], conductivity based leakage tester [24], and water hang tester [25] are among the available solutions to test glove leakage. The glove leakage test apparatus is the device that uses compressed air to pressurize the glove in order to pinpoint the exact area of the leakage by immersing the pressured glove in water. Despite the device's ability to detect holes as small as 10 μm in diameter, there is a safety risk due to the use of compressed air, as well as its weight [21]. A glove leakage tester by water level is a device that fills the glove with the necessary amount of water for hole identification. However, it is expensive and not easily affordable [22]. Because the user is exposed to ammonia gas during the test procedure, the ammonia leak testing method poses a safety risk [23]. There are three methods to test the compliance of condoms using water leakage: the Visual Leaks Method (Hang and Roll), the Conductivity Leaks Method, and the Hang and Squeeze Method [24,25]. In general, user safety issues [26,27], and expensiveness [[28], [29], [30]] are the main limitations on the existing systems to test glove and condom leakage.

Assuring the quality of gloves and condoms is a mandatory and crucial task before distribution to the end users. In this research, an integrated glove and condom leakage testing device used to detect pin holes and leaks on gloves and condoms is proposed. The device uses a specified water level based on ISO standards in which a sample of gloves and condoms is filled with water automatically using a water pump. The device is important to ensure the safety of healthcare workers, patients, and the general public in resource poor settings.

## 2 Material and methods

### 2.1 The proposed design

**Fig. 1:**
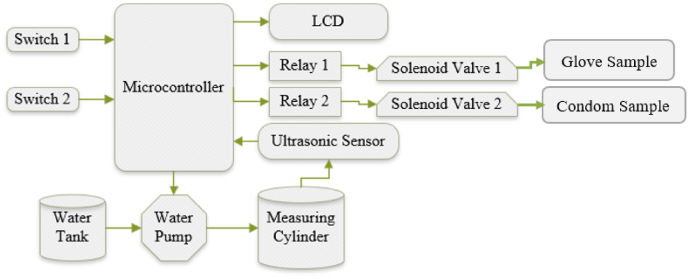
Block diagram of low-cost glove and condom leak test device.

The proposed low-cost device for testing glove and condom leakage has both a glove holder and a condom holder that allows simultaneous testing of glove and condom. Two manual switches (Switch 1 for the glove and Switch 2 for the condom) are used to initiate the automatic filling of the required volume of water in the glove and condom under investigation. The 5-V direct current (DC) pump is located in the water tank to pump the water to the measuring cylinder. The automatic measurement of water in the measuring cylinder will be achieved with the help of an ultrasound sensor. The ultrasound sensor measures the distance between the water's surface and the sensor. Then the height of the cylinder filled with water can be easily calculated by subtracting the distance measured by the sensor from the total height of the measuring cylinder. Finally, the volume is calculated by multiplying the height by the surface area of the measuring cylinder. Two electrical relays (automatic switches) are needed to activate the solenoid valve connected to the glove line and condom line. The solenoid valves open once the required volume of water for testing purposes is accumulated in the measuring cylinder so that the water gets transferred to the glove or condom connected to the device. A liquid crystal display (LCD) is used to display the measured volume of water for the end user, while a microcontroller is used to control the overall system function based on the program code. Fig. 1 shows the general block diagram of the proposed system.

### 2.2 Glove leakage testing method

When the user turns on switch 1 as shown in Fig. 1, the pump begins to fill the graduated cylinder by pumping water from the water tank. An ultrasonic sensor mounted on the top of the graduated cylinder continuously measures the amount of water, so the system automatically turns off the water pump when the water in the graduated cylinder reaches 1000 ml. The system then activates solenoid valve 1 via relay 1 to allow water in the graduated cylinder to enter the glove sample connected to the glove holder. Once water enters the glove sample, the user performs a visual test by squeezing the glove filled with water to check for leaks.

### 2.3 Condom leakage testing method

When the user turns on switch 2 as shown in Fig. 1, the water pump begins to fill the graduated cylinder. Again, the same ultrasonic sensor mounted on top of the graduated cylinder continuously measures the amount of water, so the system automatically turns off the water pump when the graduated cylinder reaches 300 ml of water. The system then activates solenoid valve 2 via relay 2 to allow water in the graduated cylinder to enter the condom sample connected to the condom holder. Once the water enters the condom sample, the user performs a visual test by squeezing the water-filled condom at three different points such as at the tip, middle and upper part of the condom to check for leaks.

### 2.4 Simulation

**Fig. 2:**
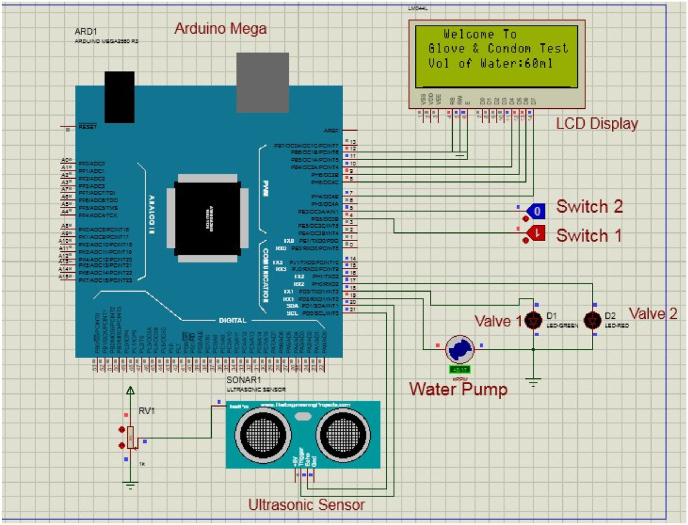
The result of the controlling system simulation.

Simulation of the controlling part was mandatory before the construction of the actual device prototype. During this simulation, the system's response to user input to automatically fill the glove and condom under investigation with the required volume of water was successfully implemented and tested. The simulation was performed on Proteus software version 8.0, developed by Labcenter Electronics Ltd. in the United Kingdom. The program code was developed using the Arduino IDE (Integrated Development Environment), an open-source Arduino software. As shown in Fig. 2, switch 1 is ON, which results in the starting of the water pump until the volume of water reaches 1000 ml. For simulation purposes, light-emitting diodes are used in place of solenoid valves to indicate the valve status as open or closed. Fig. 2 shows valve 1 is in an OFF state since the volume is below 1000 ml.

### 2.5 Final prototype

**Fig. 3:**
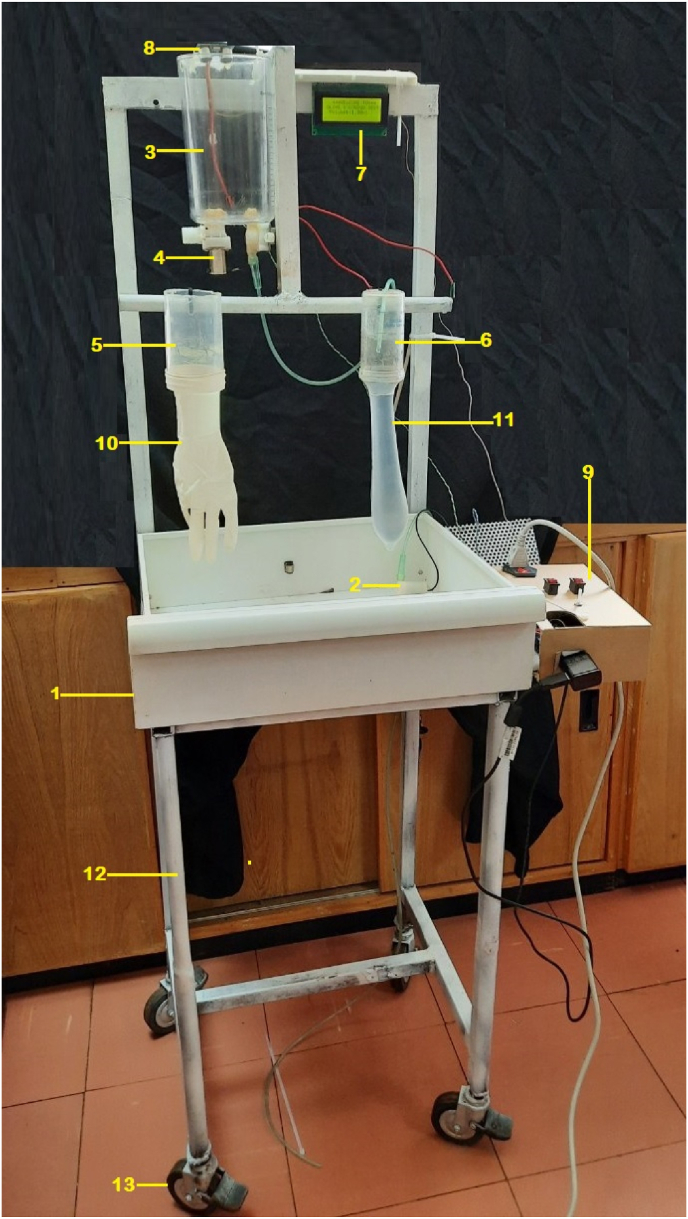
Final prototype of low-cost glove and condom leakage tester.

The final prototype of the proposed device was constructed as shown in Fig. 3. The electronic parts were tested at different stages before final integration. These tests include the reading of an ultrasonic sensor, LCD, the functionality of relays and solenoid valves. The regulated direct current (DC) power supply with an output of 12 V and 5 V was developed to supply power for solenoid valves, pumps, the Arduino Mega microcontroller, and other electronic components. The water container was made of galvanized metal with the dimensions of 40 cm × 45 cm x 15 cm in length, width, and height, respectively. For the measuring cylinder, a commercially available clear acrylic plastic bottle with a height of 19 cm and a diameter of 11.5 cm was used. Plastic tubes with a diameter of 8 cm and 5 cm for glove and condom holders were implemented, respectively. The overall frame of the device was made of a square metal frame with dimensions of 2 cm × 2 cm. The water container was fixed at a distance of 80 cm from the ground on the frame to allow the user to perform testing of samples in the sitting position comfortably. Fig. 3 clearly shows different parts of the final prototype constructed: water container [1], water pump [2], measuring cylinder [3], solenoid valve [4], glove holder [5], condom holder [6], LCD [7], Ultrasonic sensor [8], control unit [9], glove sample [10], condom sample [11], device frame [12] and castor wheel [13]. The control board [9] houses the control circuit, including the power supply, relays, and switches.

The materials used for the construction of the electrical and control systems of the final prototype are listed in Table 1 with their specifications.

**Table 1:** Materials used for construction of the prototype.

S·N	Components	Specification
1	Microcontroller	Arduino Mega 2560
2	Ultrasonic sensor	HC-SR04
3	Solenoid valve	2-way, 0.5-inch diameter, 12 V
4	Relay	5 V
5	Switch	10A, 125 V
6	LCD	20 × 4 Alphanumeric
7	Water pump	5 V
9	Transformer	220 V to 12-V step down transformer
10	Diode	1N5408 rectifier diode
11	Voltage regulator	7805 and 7812
12	Capacitor	2200 μF
13	Resistors	10 kΩ
14	Potentiometer	10 kΩ
15	Wires	Male and female jumper wires

## 3 Results

The accuracy of water volume measurement by ultrasonic sensor was evaluated by performing the test over 10 iterations for both condom and glove samples as shown in Table 2. The percentage error for the measurement of water volume for condom samples and glove samples was computed using Equation (1) from Ref. [28].

(1) $$\%\;\mathrm{ErrorVolume}=\left(\frac{\Delta \mathrm{Volume}\left(\mathrm{ml}\right)}{\mathrm{Measuring}\;\mathrm{Cylinder}\;\mathrm{Volume}\;\left(\mathrm{ml}\right)}\right)\;x\;100\%$$

**Table 2:** Performance evaluation of our device with standard measurement.

Iteration number	Volume measurement test for Condom sample			Volume measurement test for Glove sample		
	Using a measuring cylinder (in ml)	Using our Device (in ml)	% Error	Using a measuring cylinder (in ml)	Using our Device (in ml)	% Error
1	295	300	1.69	994	1000	0.60
2	296	300	1.33	992	1000	0.81
3	296	300	1.33	995	1000	0.50
4	298	300	0.67	993	1000	0.70
5	296	300	1.33	991	1000	0.91
6	295	300	1.67	994	1000	0.60
7	297	300	1.00	992	1000	0.81
8	295	300	1.67	993	1000	0.70
9	296	300	1.33	994	1000	0.60
10	296	300	1.33	992	1000	0.81
**Average % Error**			**1.34**			**0.71**

The minimum percent error obtained for volume measurement for the condom test was 0.67% and the maximum was 1.69%. The water volume measurement test for glove samples yields a minimum percent error of 0.6% and a maximum percent error of 0.91%. Accordingly, accuracy of 98.66% and 99.29% was achieved for the condom sample and glove sample, respectively. The other design criteria considered for the test were user safety, cost-effectiveness, user friendliness, and portability. The safety of the device was evaluated based on the ISO quality standard for measurement and by comparing it with other existing solutions. Accordingly, the device is safe to be used by the operator since there is no pressurizing system and water is used as a test solution. It takes 2–3 min to test one sample by a first-time user after training. Development of the device at a low cost was a focal point of this research. The cost of the materials used to develop the prototype was calculated and compared with existing devices. Accordingly, an overall cost of $104.49 was used to develop the prototype, which is much lower than the cost of existing devices. The overall weight of our device is less than 10 kg. The detailed test performed on our device is summarized in Table 3.

**Table 3:** Results of tests formed on our device.

Design Criteria	Test methodology	Result
Accuracy	Comparing the water volume measurement by our device with gold standard	Accuracy of 98.66% for condom sample and 99.29% for glove sample were obtained
Cost	Calculating the cost of items used to construct the prototype	$104.49
User safety	By comparing the testing method used in our device with the methods used in existing devices and ISO standards	Water is used for testing the leakage of gloves and condoms in our device. Hence it is safe for the user.
User friendliness	The time taken for a first-time user to use the device	It takes 2–3 min to test one sample by first time user
Portability	Device weight	<10 Kg

## 4 Discussion

Medical gloves and condoms should meet infection protection requirements to prevent infectious diseases that can affect health workers due to medical glove perforations, and also every individual who has sexual intercourse could be subjected to sexual transmitted diseases (STD's) because of low quality condoms.

There are some user safety issues with existing technologies to test gloves and condoms. For instance, the use of safety glass is recommended when using GLTA due to the use of compressed air [26]. ALTM, on the other hand, uses ammonia gas, which has a different effect on the health of the user [27]. But our device does not use compressed air and ammonia gas, but rather water to ensure the safety of the user. Cost is another barrier to accessing the existing technologies in resource poor settings. For instance, a glove integrity tester costs up to $5000 to $50,000 [28,29] and a GLTA costs around $1500 [28]. Some advanced devices used only for condom leakage testing cost $1000 to $3000 [30]. But our device was developed at a cost of $104.49, combining both the glove and condom leakage tests together.

In addition, our device was developed following ISO standards [22,24]. This study was limited in terms of using quality parts and the development of printed circuit boards due to the limitation of resources. The other limitation of this work is the testing of male latex condoms only. In the future, including a female condom testing feature is highly recommended.

## 5 Conclusion

The goal of reducing hospital acquired infections in healthcare workers and patients, as well as sexually transmitted diseases and unwanted pregnancy, could be achieved by implementing this device at various levels in resource poor settings. Low cost, user-friendliness, and accessibility are the main competitive advantages of our device.

### Conflict of interest

The author reports no conflicts of interest in this work.

## Sources of funding for your research

There is no fund received for this study.

## Ethical approval

This study doesn't involve patients or human subject.

## Consent

This study doesn't involve patients or human subject.

## Author contribution

M.A., M.T., M.B., M.W., T.Y., Y.G., H.A., M.U. and H.D. are involved in study design, prototype development, prototype testing and interpretation. H.D. was also supervised the study, and write the paper. All authors have read and approved the paper.

## Registration of research studies

Name of the registry:
Unique Identifying number or registration ID:
Hyperlink to your specific registration (must be publicly accessible and will be checked):

## Provenance and peer review

Not commissioned, externally peer reviewed.

## Guarantor

Hundessa Daba Nemomssa.

## Declaration of competing interest

There is no conflict of interest in this work.
